# Supplementary material for: Hippocampal subfield vulnerability to α-synuclein pathology precedes neurodegeneration and cognitive dysfunction
Source: NPJ Parkinsons Dis. 2023 Aug 29;9:125. doi: 10.1038/s41531-023-00574-1 (PMC10462636; doi:10.1038/s41531-023-00574-1)
Supplement: Supplementary file 1 — Supplemental Information [file 41531_2023_574_MOESM1_ESM.pdf]

Supplementary Information

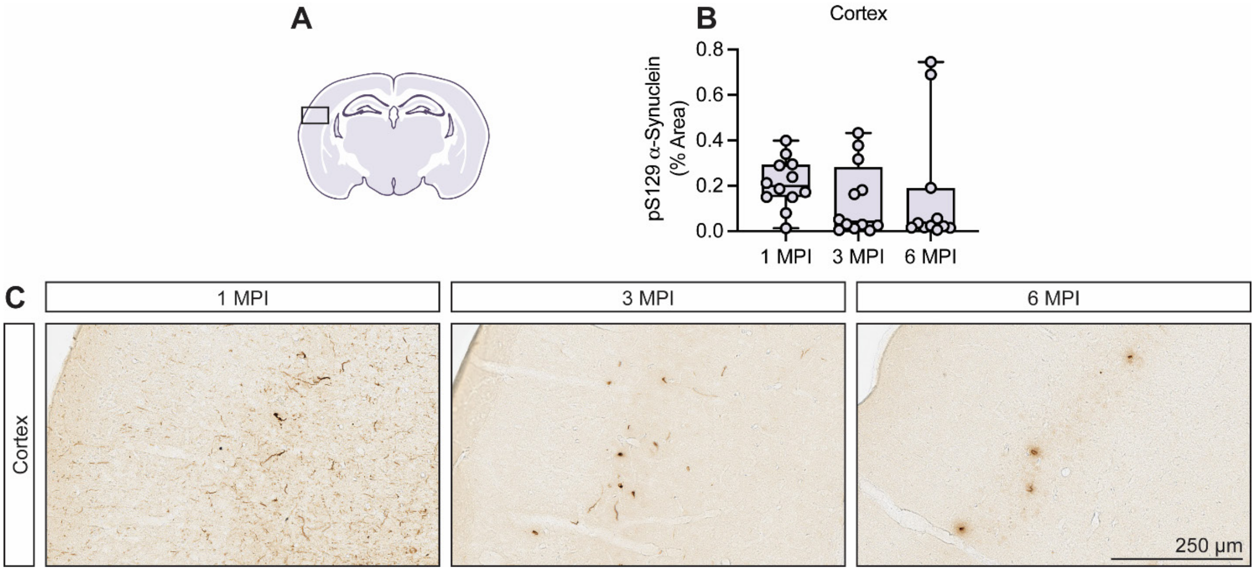

Supplementary Figure 1: Formation of α-synuclein pathology in the cortex. a) Schematic representation of the region of interest within the mouse cortex. **b)** Digital pathology quantification of pS129-α-synuclein immunostaining in the cortex at 1, 3, and 6 MPI. Data are expressed as boxplots depicting the median, interquartile range, and individual data points of % area occupied of pS129-α-synuclein immunostaining (*n* = 11-12 animals/group/timepoint). Non-significant by Kruskal-Wallis test with Dunn's multiple comparison test. **c)** Representative images of pS129-α-synuclein immunostaining at 1, 3, and 6 MPI in the cortex. Scale bar: 250 µm.

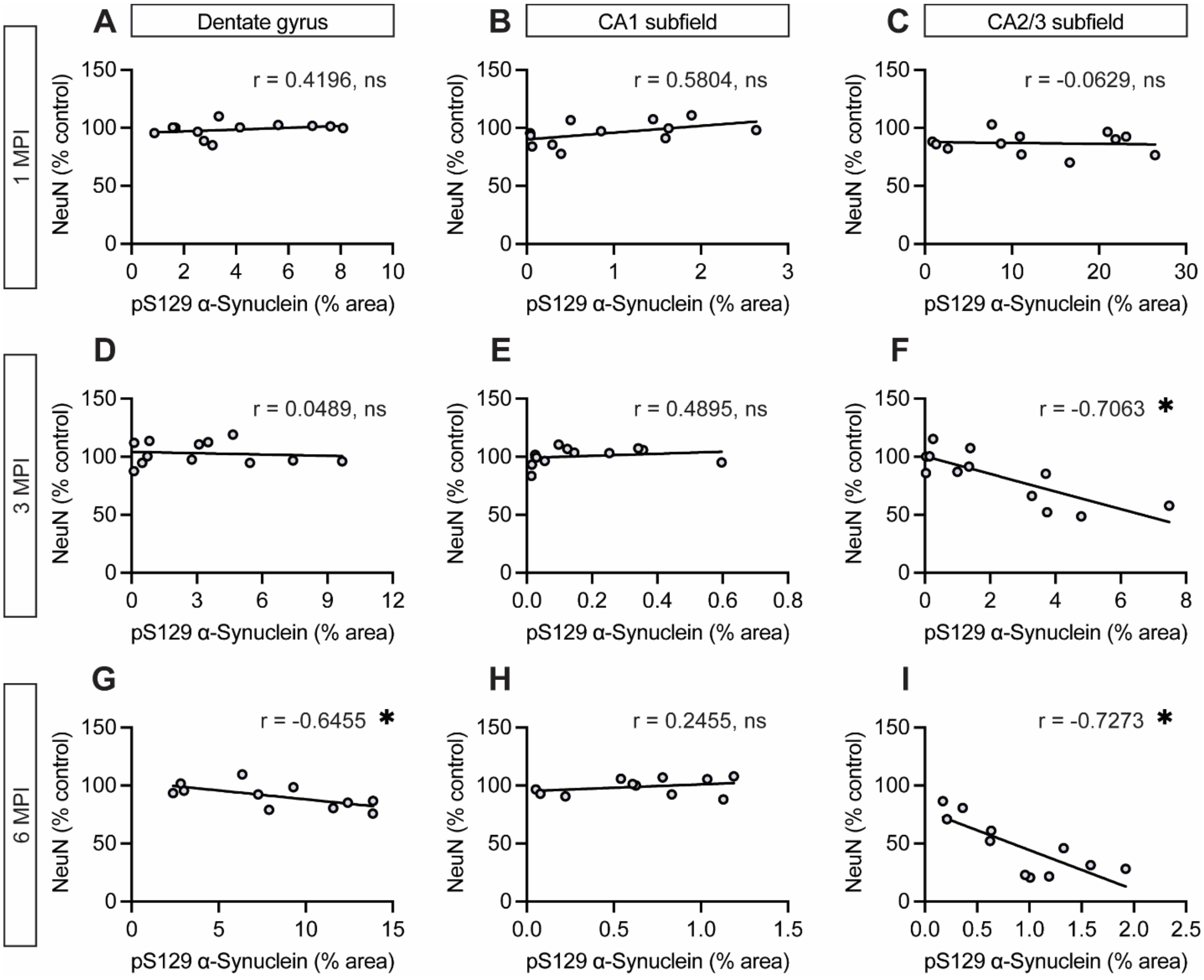

Supplementary Figure 2: Relationship between neuronal loss and residual α-synuclein pathology in the hippocampus. a-i) Association between normalized NeuN levels and pS129-α-synuclein immunostaining in the **(a, d, g)** dentate gyrus, **(b, e, h)** CA1 subfield, and **(c, f, i)** CA2/3 subfield at **(a, b, c)** 1 MPI, **(d, e, f)** 3 MPI, and **(g, h, i)** 6 MPI (*n* = 11-12 animals/group). Data are expressed as individual data points. **P*<0.05 by Spearman correlation, *r* values listed by region, as indicated.

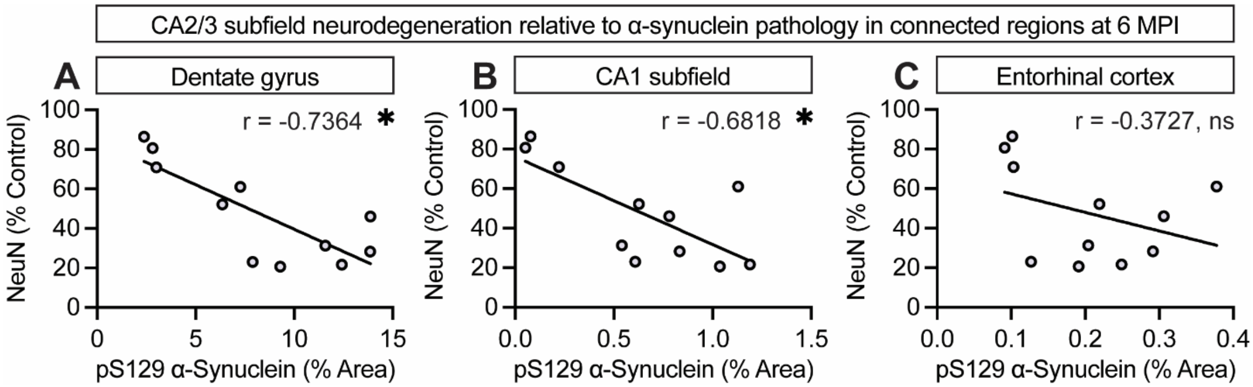

Supplementary Figure 3: Spread of α-synuclein pathology into the dentate gyrus and CA1 subfield is associated with neuronal loss in the CA2/3 subfield. a-c) Association between CA2/3 subfield NeuN levels and pS129-α-synuclein immunostaining in adjacent regions at 6 MPI including **(a)** dentate gyrus, **(b)** CA1 subfield, and **(c)** entorhinal cortex. **P*<0.05 by Spearman correlation, *r* values listed by region, as indicated.

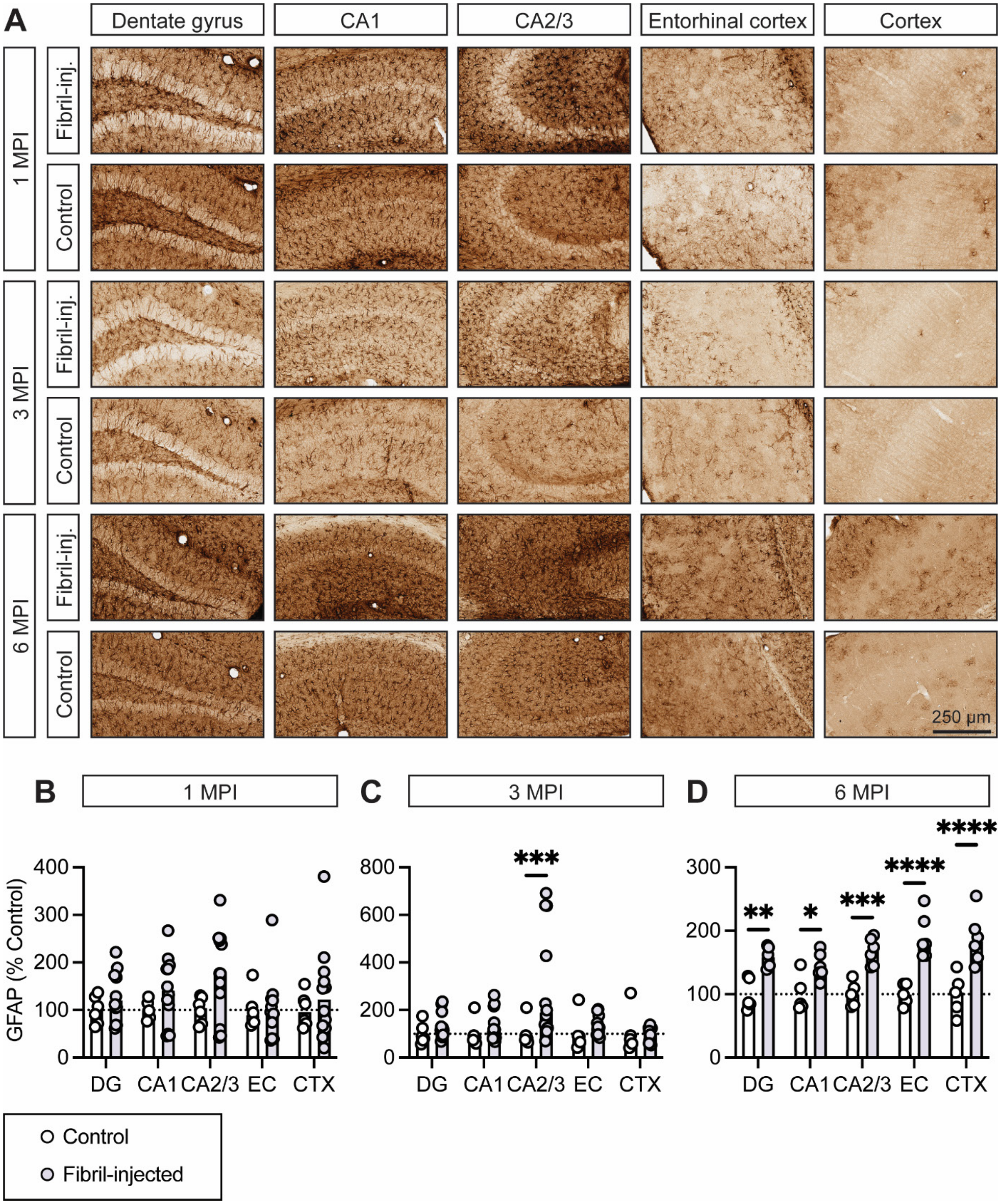

Supplementary Figure 4: Astrogliosis is detected in fibril-injected mice. a) Representative images of GFAP immunostaining at 1, 3, and 6 MPI in the hippocampal subfields and cortical regions, arranged by fibril-injected and control group. **b-d)** Digital

pathology quantification of GFAP immunostaining by region at 1, 3, and 6 MPI. Data are expressed as boxplots depicting the median, interquartile range, and individual data points of % area occupied of GFAP immunostaining normalized to control at **(b)** 1 MPI, **(c)** 3 MPI, and **(d)** 6 MPI (*n* = 11-12 animals/group). *P<0.05, **P<0.01, ***P<0.001, or ****P<0.0001 by two-way ANOVA with Bonnferoni's multiple comparisons test, as indicated. Scale bar: 250 μm.

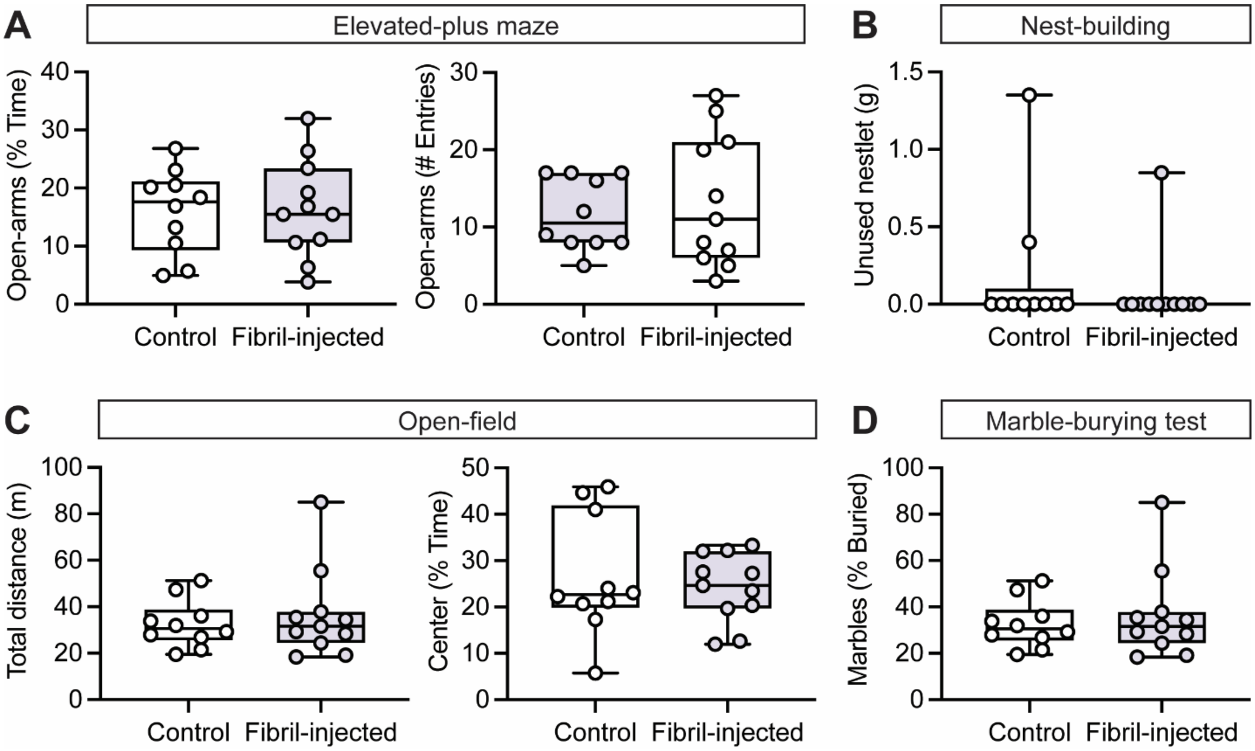

Supplementary Figure 5: Fibril-injected mice do not exhibit motor deficits or changes in anxiety-related behavior. a) Performance in the elevated plus-maze as measured by % time in the open arms and by total number of entries into the open arms. **b)** Scoring of nest-building activity by measurement of unused nestlet material. **c)** Open-field performance measured by total distance travelled and by % time spent in the center of the apparatus. **D)** Marble-burying activity depicted by % marbles buried during assay. All assays measured at 6 MPI (*n* = 10-11 animals/group). Non-significant by unpaired Student's *t*-test.

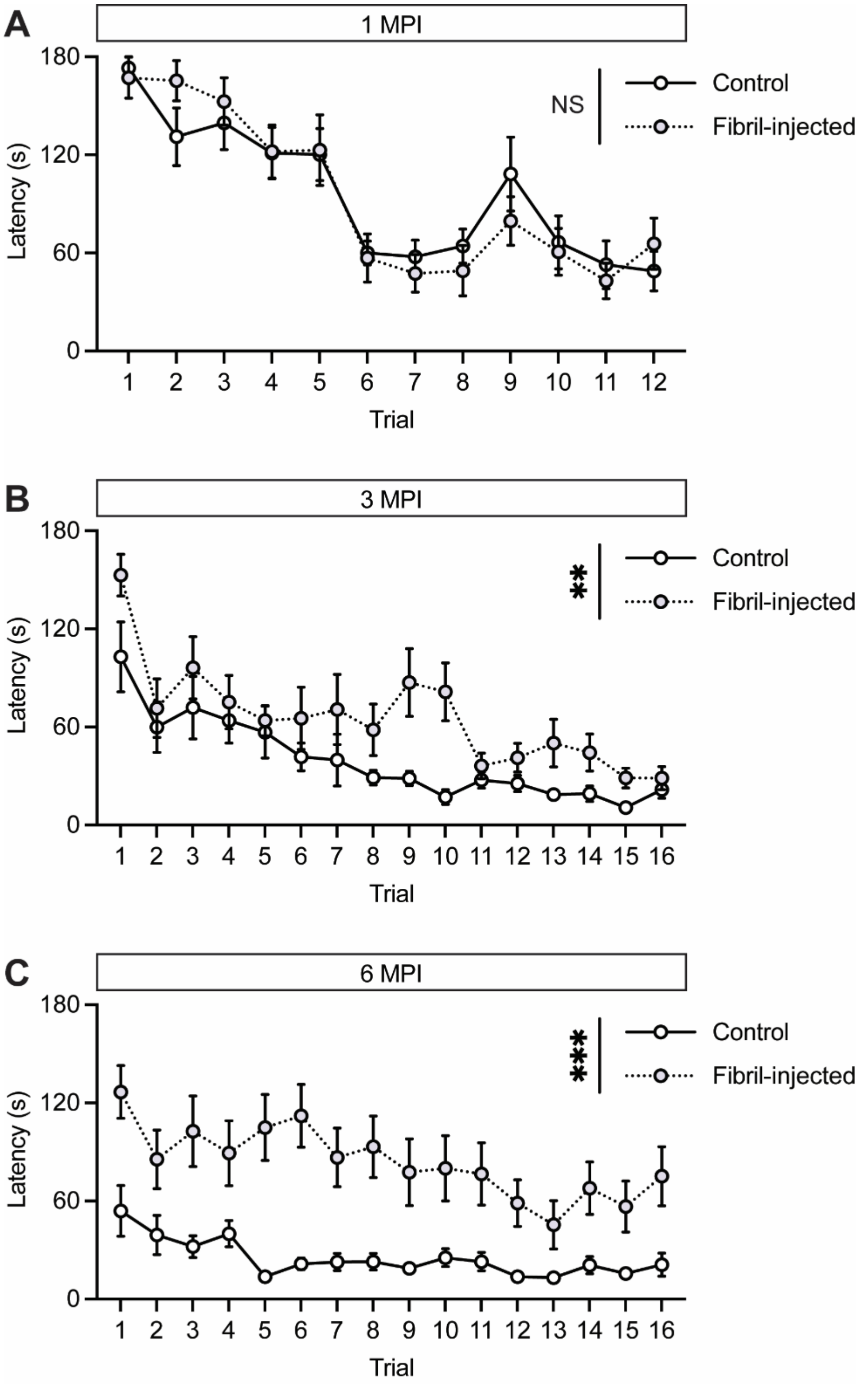

Supplementary Figure 6: Inter-trial performance in the Barnes maze at 1, 3, and 6 MPI. a-c) Barnes maze performance as measured by latency to reach target hole across sequential trials at **(a)** 1 MPI, **(b)** 3 MPI, and **(c)** 6 MPI (*n* = 10-11 animals/group). ***P*<0.01 or ****P*<0.001 by two-way ANOVA with Bonnferoni's multiple comparisons test, as indicated.

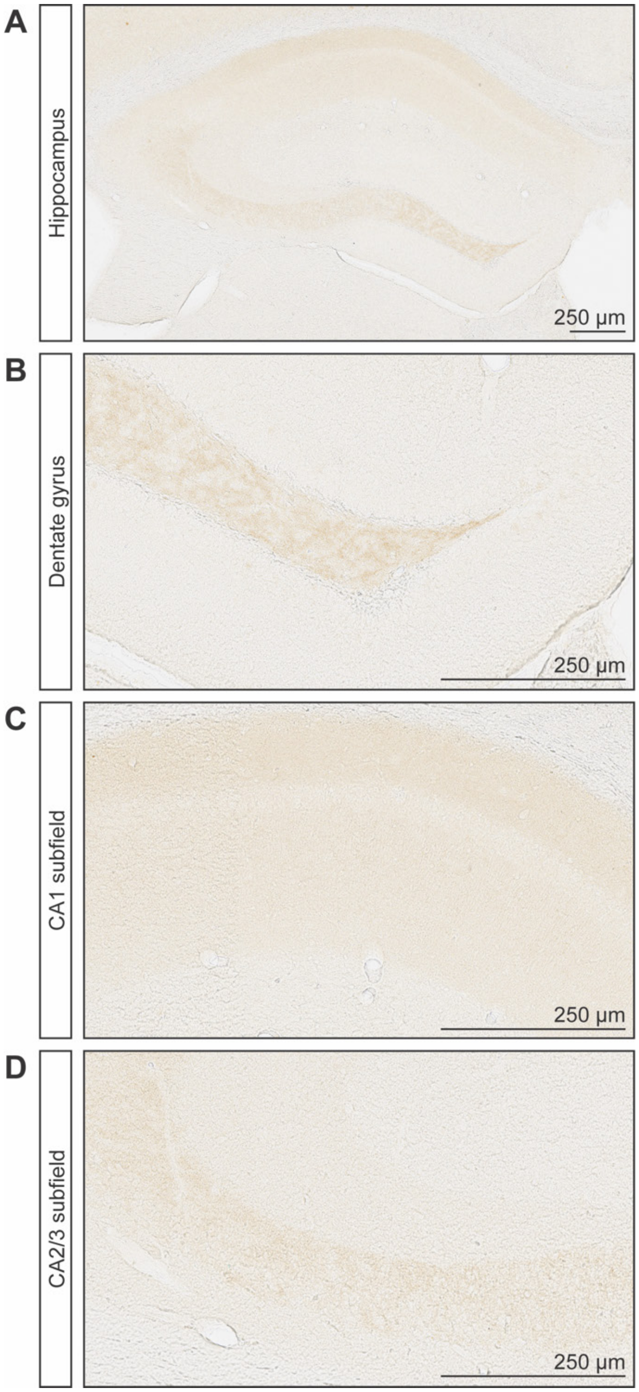

Supplementary Figure 7: Absence of pS129-α-synuclein pathology in sham-injected mice. a) Representative image of pS129-α-synuclein immunostaining in the hippocampus of sham-injected (PBS) mice at 6 MPI. **b-d)** Higher magnification images of the (**b**) denate gyrus, (**c**) CA1 subfield and (**d**) CA2/3 subfield depicting and absence of inclusion pathology. Scale bars: 250 µm.
